# Inhibition of apoptosis in neuronal cells infected with *Chlamydophila (Chlamydia) pneumoniae*

**DOI:** 10.1186/1471-2202-9-13

**Published:** 2008-01-24

**Authors:** Denah M Appelt, Maria R Roupas, Deana S Way, Marcus G Bell, Elizabeth V Albert, Christine J Hammond, Brian J Balin

**Affiliations:** 1 Department of Neuroscience, Physiology & Pharmacology, Philadelphia College of Osteopathic Medicine, Philadelphia, USA; 2 Department of Pathology, Microbiology & Immunology, Philadelphia College of Osteopathic Medicine, Philadelphia, USA; 3 Center for Chronic Disorders of Aging; Philadelphia College of Osteopathic Medicine, Philadelphia, USA

## Abstract

**Background:**

*Chlamydophila *(*Chlamydia*) *pneumoniae *is an intracellular bacterium that has been identified within cells in areas of neuropathology found in Alzheimer disease (AD), including endothelia, glia, and neurons. Depending on the cell type of the host, infection by *C. pneumoniae *has been shown to influence apoptotic pathways in both pro- and anti-apoptotic fashions. We have hypothesized that persistent chlamydial infection of neurons may be an important mediator of the characteristic neuropathology observed in AD brains. Chronic and/or persistent infection of neuronal cells with *C. pneumoniae *in the AD brain may affect apoptosis in cells containing chlamydial inclusions.

**Results:**

SK-N-MC neuroblastoma cells were infected with the respiratory strain of *C. pneumoniae*, AR39 at an MOI of 1. Following infection, the cells were either untreated or treated with staurosporine and then examined for apoptosis by labeling for nuclear fragmentation, caspase activity, and membrane inversion as indicated by annexin V staining. *C. pneumoniae *infection was maintained through 10 days post-infection. At 3 and 10 days post-infection, the infected cell cultures appeared to inhibit or were resistant to the apoptotic process when induced by staurosporine. This inhibition was demonstrated quantitatively by nuclear profile counts and caspase 3/7 activity measurements.

**Conclusion:**

These data suggest that *C. pneumoniae *can sustain a chronic infection in neuronal cells by interfering with apoptosis, which may contribute to chronic inflammation in the AD brain.

## Background

The mechanisms and morphological changes that underlie the apoptotic process leading to cell death have been well-described [1,2]. In this regard, the induction of apoptosis has been shown to be important in embryonic development and in defending against infection of a eukaryotic host by microorganisms. Examples of the latter have been shown for both viral and bacterial infections [3,4]. In contrast, some organisms, such as the intracellular pathogen *Chlamydia pneumoniae*, have the capacity to inhibit the apoptotic process following infection [5-7]. This inhibition has been demonstrated in a number of different cell types, including neutrophils [8], monocytes [9,10], epithelial cells [10-12], and microglial cells [13]. Some *Chlamydia-*infected host cells are resistant to pro-apoptotic stimuli such as TNFα, Fas antibody, staurosporine, and UV-light [6,14], and *C. pneumoniae *infection has been shown to down regulate pro-apoptotic cytoplasmic proteins such as caspase-3 and cytochrome c. Intriguingly, *C. pneumoniae *infection also has been shown to activate anti-apoptotic proteins such as Bcl-2 and NF-κβ [15], the latter of which is critical in the expression of multiple genes involved in inflammatory responses and anti-apoptotic mechanisms [16].

Our laboratory has demonstrated *C. pneumoniae *infection in brain tissues from patients diagnosed with AD [17,18]. Many cell types including monocytes, endothelial cells, glial cells and neurons were shown to be infected. Interestingly, the infected cells did not appear to be undergoing degenerative changes, even though they were in the vicinity of cells demonstrating neurodegenerative pathology characteristic of AD. *C. pneumoniae *also has been identified within neurons in the AD brain by in situ hybridization [19] as well as in the olfactory neuroepithelia, the olfactory bulbs and endothelia from *C. pneumoniae*-infected BALB/c mice by immunohistochemistry [20]. Collectively, these studies have correlated infection with *C. pneumoniae *to the neuropathology characteristic of AD, but the specific influences of infection on neuronal cell injury, cell death, and chronic inflammation are still being determined.

A major factor in AD is an inflammatory process thought to be stimulated by the processing and deposition of β-amyloid (Aβ1–42). Although β-amyloid may promote inflammation, infectious agents such as *C. pneumoniae *also could provoke neuroinflammation in sporadic, late-onset AD that precedes or coincides with the deposition of Aβ1–42 in the AD brain. The exact role that chlamydial infection plays with respect to abnormal processing and deposition of amyloid, however, remains to be determined and is beyond the scope of this report. In contrast, the focus of the current study was to determine how *C. pneumoniae *infection influences the apoptotic process within neuronal cells in culture during both short and long term infections.

## Results

**Figure 1:**
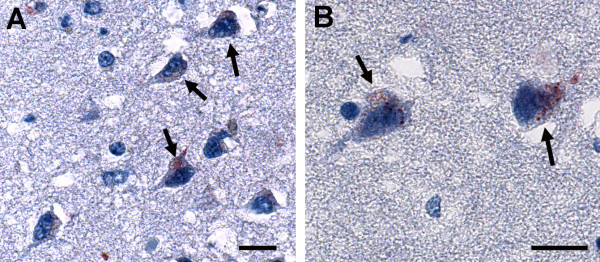
**Alzheimer diseased brain tissue from the hippocampal region**. Tissue sections were labeled with an anti-chlamydial specific antibody revealing chlamydial inclusions within neurons (arrows, panels A and B). (A & B) Mag. bars = 20 μm.

Several experimental paradigms (each repeated a minimum of 3 times) were utilized in these studies to determine the relationship between *C. pneumoniae *infection and apoptosis in neuronal cells as it relates to the pathogenesis of AD. *C. pneumoniae *was identified within neurons in hippocampal AD brain tissues (Figure 1). Immunolabeling using antibodies specific to *C. pneumoniae *revealed a punctate labeling pattern identifying the chlamydial bodies within several neurons (Figure 1). Delineation of the chlamydial bodies from lipofuscin in the neurons was determined using a secondary antibody conjugated to alkaline phosphatase red as compared to the substrate diaminobenzine (DAB), since lipofuscin and DAB typically have a brownish appearance in histological preparations.

**Figure 2:**
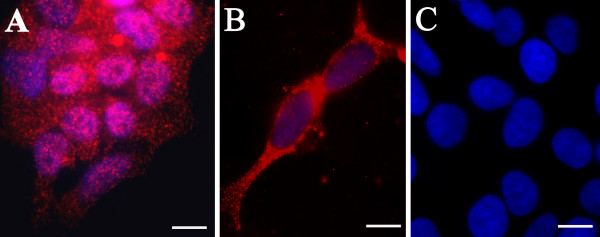
**SK-N-MC neuroblastoma cells immunolabeled with neuronal specific antibodies**. In panels A and B, neuronal phenotype was determined by labeling with anti-βIII-tubulin (red). In panel C, the cells were incubated in 0.1% DMSO in growth media for 4 hr and Hoechst-stained to highlight nuclear profiles (blue). Mag. bars = 10 μm.

To confirm that a homogenous neuronal phenotype was maintained in culture, SK-N-MC neuroblastoma cells were screened with antibodies to βIII-tubulin (Figures 2A, B), a cytoskeletal protein expressed in neuronal cells. Quantitation yielded a homogeneous neuronal phenotype; greater than 90% of the cells labeled for βIII-tubulin. These cells were also analyzed for any morphological changes that may occur following incubation with 0.1% dimethylsulfoxide (DMSO), the vehicle for the apoptotic inducing agent staurosporine, as DMSO has been used at higher concentrations to induce apoptosis in cultured cells. In these control experiments, the cell's nuclear integrity did not reveal any morphological changes characteristic of apoptotic nuclear fragmentation as is evident from the Hoechst-stained nuclear profiles (Figure 2C). Additionally, when cells were incubated with the FITC- or rhodamine-conjugated goat anti-mouse secondary antibody without prior labeling by primary antibody, non-specific binding of either the FITC- or rhodamine-conjugated secondary was not observed (data not shown); this confirmed the specificity of the secondary antibodies.

**Figure 3:**
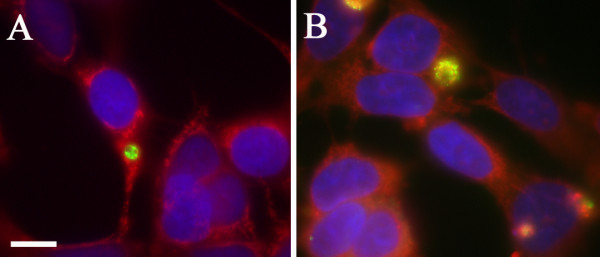
**Infection of neuronal cells followed by staurosporine incubation**. SK-N-MC neuroblastoma cells infected with *Chlamydia pneumoniae *for 3 days (A) and 10 days (B), revealing chlamydial inclusions (green). In panel A, Imagen anti-chlamydial specific antibody was used. In panel B, anti-chlamydial antibodies 60C19 and Imagen were used in combination to detect prolonged or persistent infection. Nuclei were identified by Hoechst stain (blue), and the cytoplasm was stained with Evans blue (red). Mag. bar = 10 μm.

In order to determine the effects exerted on the apoptotic process by an infection with *C. pneumoniae *in neuronal cells, SKNMC neuroblastoma cells were inoculated with the respiratory strain of *C. pneumoniae*, AR-39, at an MOI of 1. These cells were propagated for 3 and 10 days post-infection followed by immunolabeling with FITC-conjugated anti-chlamydial antibodies, 60C19 and Imagen containing Evans blue staining the cytoplasm red (Figure 3). At both 3 and 10 days post-infection chlamydial inclusions were observed and the nuclei did not appear fragmented as revealed by Hoechst staining.

**Figure 4:**
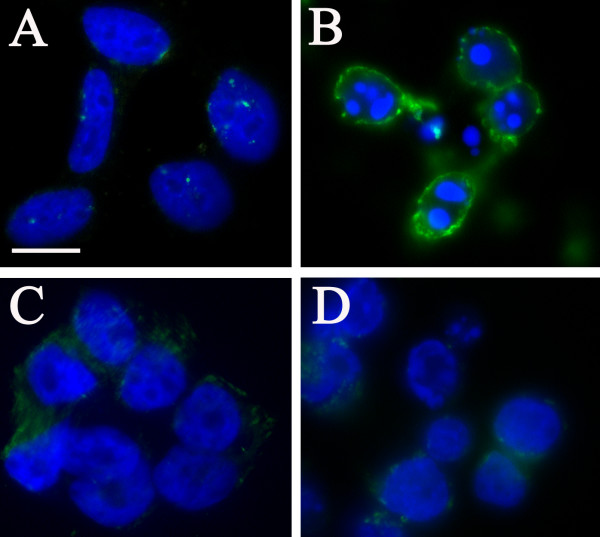
**Annexin labeling of SK-N-MC neruoblastoma cells infected with *Chlamydia pneumoniae *following staurosporine incubation**. SK-N-MC neuroblastoma cells uninfected (A, B) and infected with *Chlamydia pneumoniae *for 3 days (C, D), untreated cells (A, C) and cells treated with 1 μM staurosporine for 4 hours (B, D), labeled with annexin V (green) and DAPI (blue). Mag. bar = 10 μm.

During apoptosis, phosphatidylserine is translocated from the inner to the outer plasma membrane leaflet. This externalization was analyzed with annexin V-FITC in conjunction with Hoechst staining to examine nuclear fragmentation in 3 day infected cells. In the absence of staurosporine, uninfected cells (Figure 4A) and those infected with *C. pneumoniae *(Figure 4C) showed no evidence of apoptosis by either Hoechst or annexin V staining. Staurosporine-treated, uninfected cells, however, showed prominent nuclear fragmentation and phosphatidylserine externalization (Figure 4B). In the case of staurosporine-treated, *Chlamydia*-infected neuroblastoma cells, most nuclear profiles appeared unaffected with only a few cells demonstrating very early apoptotic morphologic changes (Figure 4D).

**Figure 5:**
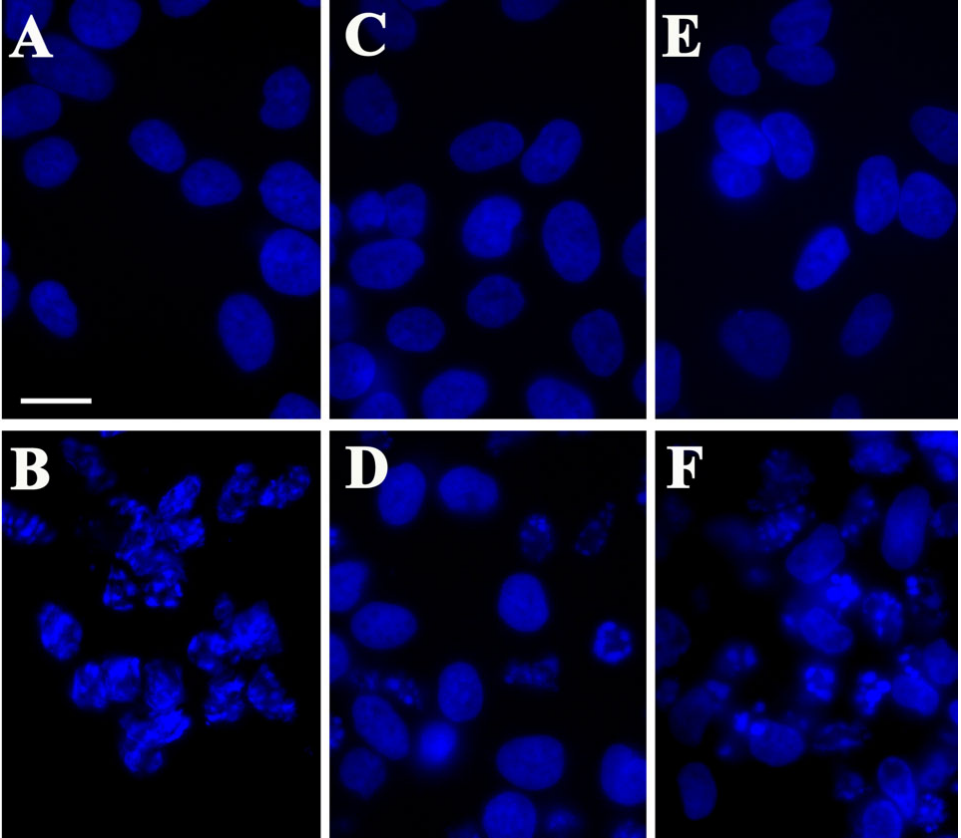
**Analysis of the nuclear profiles of *Chlamydia pneumoniae*-infected neuroblastoma cells following apoptotic induction with staurosporine**. Nuclear profiles were revealed with Hoechst stain and counted as normal or apoptotic depending on fragmentation. Conditions in panels A-F are as follows: A, uninfected cells; B, uninfected cells treated with 1 μM staurosporine for 4 hours; C,: cells infected for 3 days; D, cells infected for 3 days treated with 1 μM staurosporine for 4 hours; E, cells infected for 10 days; F, cells infected for 10 days treated with 1 μM staurosporine for 4 hours. Mag. bar = 20 μm.

**Figure 6:**
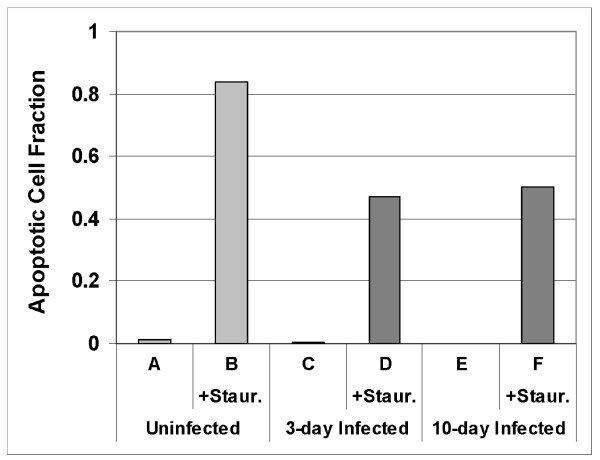
**Quantitative analysis of nuclear profile data**. Lanes A-F correspond to the conditions in panels A-F of figure 5. Data are from 3 independent infections with 20 random fields counted for each condition. Total cell counts are: A, 793; B, 436; C, 512; D, 412; E, 402; F, 395. Chi-squared analysis of these data indicates that the difference between the uninfected result and the infected results (either 3 or 10 days) is highly statistically significant (*p *< 0.0001), however the infected results were not significantly different from each other (*p *= 0.36)

Hoechst staining was used to visualize the nuclear morphology of neuroblastoma cells in which uninfected cells and cells infected with *C. pneumoniae *were induced to undergo apoptosis by staurosporine (Figure 5). Nuclear profiles were analyzed in 20 random fields from three separate experiments (Figure 6). Uninfected cells (Figures 5A and 6A), cells infected for 3 days (Figures 5C and 6C) and cells infected for 10 days (Figures 5E and 6E) that had not been exposed to staurosporine yielded minimal nuclear fragmentation. When treated with staurosporine, 84% of uninfected neuroblastoma cells demonstrated apoptotic nuclear profiles (Figures 5B and 6B). In contrast, nuclear fragmentation characteristic of apoptosis was observed in only 47% of cells that were infected for 3 days (Figures 5D and 6D) and 50% of cells infected for 10 days (Figures 5F and 6F) when subsequently treated with staurosporine. Chi-squared analysis indicated that this inhibition of apoptosis by the infection was highly statistically significant (p < 0.0001).

**Figure 7:**
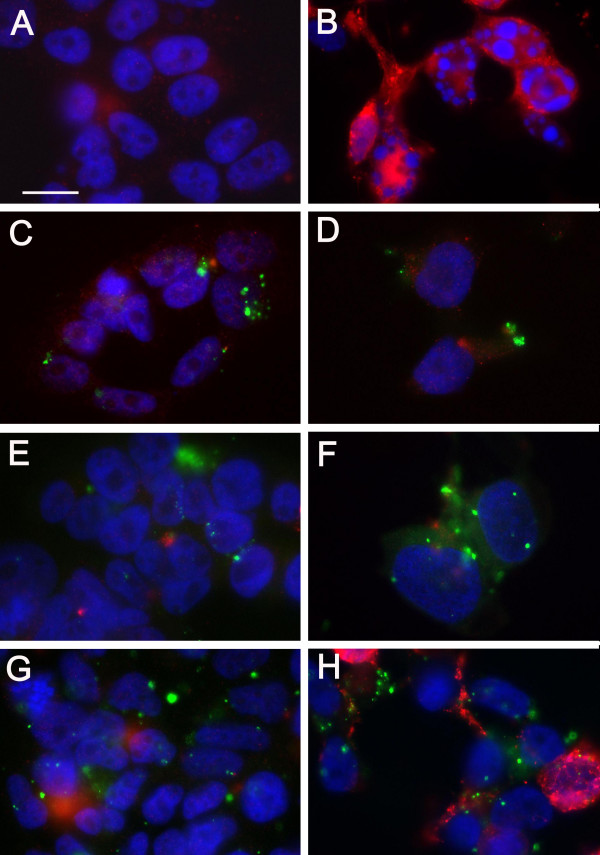
**SK-N-MC neuroblastoma cells immunolabeled for active caspase 3 and C. pneumoniae inclusions following induction of apoptosis with staurosporine**. Anti-caspase 3 specific for activated caspase 3 (red); DAPI to reveal the nuclear profile (blue); anti-chlamydial antibody specific for chlamydial inclusions (green). Shown are uninfected (A, B) and infected cells for 24 (C, D), 48 (E, F) and 72 (G, H) hours post infection, untreated cells (A, C, E, G) and cells treated with 1 μM staurosporine for 4 hours (B, D, F, H). Mag. bar = 20 μm.

The effect of infection by *C. pneumoniae *upon the caspase pathway in the apoptotic process was examined using immunocytochemistry techniques. SK-N-MC neuronal cells were infected with the AR39 strain of *C. pneumoniae *and incubated with 1 μM staurosporine for 4 hours to induce apoptosis (Figure 7), then screened for apoptosis with an antibody specific for the activated form of caspase 3, in conjunction with Hoechst staining to reveal the nuclear profiles. In the absence of staurosporine, uninfected SK-N-MC cells revealed a normal nuclear profile (Figure 7A) with minimal active caspase-3, whereas uninfected cells treated with staurosporine clearly demonstrated the characteristic apoptotic nuclear fragmentation and activated caspase 3 (Figure 7B). In cells infected with *C. pneumoniae *(panels C-H), inclusion bodies were detected by immunolabeling with a specific anti-chlamydial antibody. In non-staurosporine treated cells at 24, 48 and 72 hrs post-infection, there was virtually no visual evidence of apoptosis (Figure 7, panels C, E, G). In contrast, in cells infected with *C. pneumoniae* and treated with staurosporine (Figure 7, panels D, F, H), only in the 72 hr post-infection culture was there limited evidence of apoptotic nuclear fragmentation and activated caspase 3 (Figure 7, panel H).

**Figure 8:**
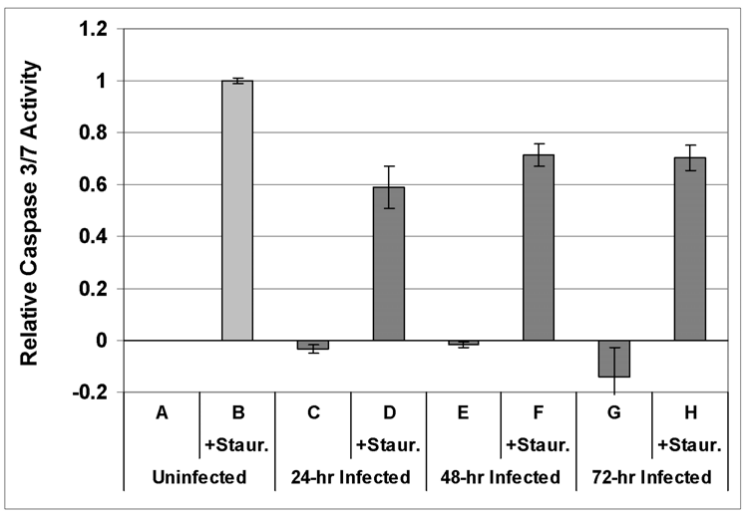
**Caspase 3/7 activity detected by the Apo-ONE assay**. Plotted relative to the increase in activity in uninfected cells induced by staurosporine, at three timepoints after infection with *C. pneumoniae*. Lanes A-H correspond to the conditions in panels A-H of figure 7. Lanes C, E and G depict decreased activity relative to uninfected control. Error bars are s.e.m. for 9 samples of 10^4 ^cells from 3 cell culture preparations.

To assess whether caspase activity is inhibited by *C. pneumoniae *in acute or extended stages of infection, caspase activity was quantitatively measured for uninfected cells and cells at 24, 48 and 72 hours post-infection. The change in caspase 3/7 activity induced by 1 μM staurosporine is plotted in Figure 8 relative to the change observed in uninfected cells (lanes A and B). In cells assayed at 24 hours post-infection (lanes C and D), caspase 3/7 activity in the absence of staurosporine was slightly suppressed compared to uninfected cells, but most notably the increase in activity induced by staurosporine was suppressed to 0.59 ± 0.08 compared to staurosporine-treated, uninfected cells (1.00 ± 0.01). At longer times post-infection, staurosporine induced a larger increase in activity (0.72 ± 0.04 at 48 hr p.i., 0.70 ± 0.05 at 72 hr p.i., lanes F and H respectively), indicating that the infection was less inhibitory at these time points, most likely as a result of a decrease in the fraction of infected cells in the cultures at these longer infection times.

## Discussion

The current *in vitro *neuronal studies demonstrate that infection of human neuroblastoma cells by C.pneumoniae has an effect on apoptosis following staurosporine induction, as measured by characteristics of apoptosis such as nuclear fragmentation, cytoplasmic membrane inversion, and caspase 3/7 activation. The data suggest that neuronal cells can develop and maintain a chronic and prolonged infection with C.pneumoniae through 10 days post-infection. Inhibition of apoptosis was measured from 24 hours through 10 days post-infection, and apoptosis inhibition was observed throughout this period. However, C. pneumoniae had a more robust effect on inhibiting apoptosis in neuronal cells at 24 hr post-infection as compared to 10 day post-infection. These results are consistent with other studies that have determined that C. pneumoniae infection inhibits apoptosis in monocytes [9,10], neutrophils [8], and epithelial cells [10-12].

Human neuroblastoma cell lines can be induced to undergo apoptosis when incubated in staurosporine at several concentrations [21]. Staurosporine is a potent inhibitor of numerous kinases, including protein kinase C and cAMP-dependent protein kinases [22], calmodulin-dependent protein kinase [23], and receptor tyrosine kinases [24]. In cells undergoing staurosporine-induced apoptosis, levels of the pro-apoptotic protein Bax at the mitochondrion are increased, resulting in release of cytochrome c and subsequent activation of caspase 9 and caspase 3 [25,26]. This release has been shown to be blocked upon infection with *C. pneumoniae *[6]. In our studies, caspase 3/7 activity was inhibited upon infection with *C. pneumoniae*, possibly resulting from inhibition of cytochrome c release. Alternatively, activation of caspase 3/7 could be inhibited by infection with *C. pneumoniae *downstream and/or upstream of cytochrome c release, consistent with inhibition of apoptosis at several levels of the apoptotic mitochondrial pathway [5,6,11,12,14,27]. One report indicated that apoptosis of endothelial cells was indeed inhibited by infection with *C. pneumoniae*, although cell death by necrosis was not [28]. Necrosis of these cells was correlated to an increased concentration of intracellular reactive oxygen species following infection.

Interestingly, while there is evidence for the induction of apoptosis in the Alzheimer's diseased brain, data on the completion of this process are questionable [29]. In AD brains, increased levels of pro-apoptotic proteins governing mitochondrial integrity and caspase activity have been demonstrated. These data suggest that caspase activation may be a key factor in modulating the apoptotic process in neurons [30]. *In vitro *experiments have shown that β-amyloid peptides can activate the caspase cascade in neurons resulting in cell death [31-33]. However, the extent to which neurons die in AD as a result of apoptosis remains controversial [16].

Apoptosis is not necessarily congruent with a slow, progressive neurodegenerative disease. It is plausible that neuronal cell death may occur through several mechanisms even though neurodegeneration may commence along with early apoptotic events. At first glance, our infection data appear to controvert findings of significant neuronal degeneration and cell death. However, because infection with *C. pneumoniae *can both inhibit apoptosis and promote cell death by necrosis [28], and because we have continually observed *C. pneumoniae *infection in sporadic, late-onset AD brain tissues [17-19], the possibility that infection of neuronal cells in AD could lead to cell death by necrosis is compelling as this would implicate the inflammatory process. There are numerous reports of significant neuroinflammation in AD in which cell damage and death are thought to occur as the result of inflammation [34].

Because the amount of amyloid precursor protein (APP) is increased in areas of AD neuropathology, many studies have focused on the cytotoxic effects of exogenous (extracellular) β-amyloid [35]. However, the role of endogenous (intracellular) β-amyloid in the neuronal cell's viability remains a conundrum. Recently, endogenous APP has been shown to have anti-apoptotic effects on cells isolated from dorsal root ganglia [36]. Suppression of apoptosis by the neuron could be an adaptation to combat stimuli that pathologically up-regulate endogenous amyloid. These stimuli, such as perhaps infection with *C. pneumoniae*, may indirectly block death of the host cell through this anti-apoptotic effect, thereby promoting the obligate intracellular bacterium's ability to replicate within the host. While inhibiting apoptosis may be favorable to the bacterium, maintaining a chronic or prolonged infection that also up-regulates amyloid could ultimately lead to chronic disease states. The neuron's tolerance to amyloid is likely to be concentration-dependent and overwhelming amyloid production and processing may result in the amyloid pathology characteristic of AD.

## Conclusion

In summary, *C. pneumoniae *readily infects neuroblastoma cells *in vitro *and maintains a prolonged infection by inhibiting apoptosis. As *C. pneumoniae *also has been demonstrated to infect neurons in AD brains [18,19], the ability to inhibit the apoptotic process could result in long-term infection *in situ*. Inhibition of apoptosis by suppression of caspase 3/7 activity, and/or by decreasing levels of active caspase 3, may be mechanisms by which *C. pneumoniae *can sustain a prolonged infection in the host and optimize its intracellular environment. Chronic and/or prolonged infection in the AD brain may promote amyloidogenesis, neuroinflammation, and ultimately pathology found in late-onset sporadic AD. In this way, infection with *C. pneumoniae *in neuronal cells could contribute to the overall neuropathogenesis of this disease.

## Methods

### Tissue Culture

SK-N-MC neuroblastoma cells (HTB-10, American Type Culture Collection, Manassas, VA) were propagated in growth medium (GM) composed of minimum essential medium (MEM) supplemented with 2 mM L-glutamine, 1.0 mM sodium pyruvate, 0.1 mM non-essential amino acids, and 10% fetal bovine serum. Hanks balanced salt solution (HBSS) and 0.05% trypsin-EDTA were used in the standard protocol for harvesting cells. Cells were incubated at 37°C with 5% CO_2_.

### Infection in Neuronal Cells

SK-N-MC neuroblastoma cells were grown to 1 × 10^5 ^confluency in T25 flasks (Corning Inc., Corning, NY) for the capase assay or in 4 well chamber slides (Nalge Nunc International, Rochester, NY) for the immunocytochemistry analysis. The cells were inoculated with 1 × 10^5 ^IFU of AR39 strain (ATCC 53592, Manassas, VA) of *C. pneumoniae *(multiplicity of infection = 1). The flasks containing 3 ml of GM were then centrifuged in a Sorvall Legend RT at 750 × g for 30 min at 20°C, then 7 ml of GM was added, or chamber slides were centrifuged at 150 × g for 30 min at 20°C. Following centrifugation, both the flasks and the chamber slides were incubated at 37°C in 5% CO_2 _for the various time points.

### Induction of Apoptosis

Uninfected and infected SK-N-MC neuroblastoma cells grown in chamber slides were induced to undergo apoptosis using staurosporine dissolved in 0.1% dimethylsulfoxide (DMSO), final concentration 1 μM staurosporine diluted in GM and incubated for 4 hr at 37°C in 5% CO_2_. To control for possible affects that DMSO alone might have on the cell's nuclear integrity, cells were incubated in 0.1% DMSO without staurosporine. Cells were then washed with HBSS and processed for immunocytochemistry.

### Immunofluorescence (IF)

Cells processed in chamber slides were rinsed with PBS pH 7.4 followed by fixation in 100% cold MEOH for 5 min or for 30 min in 1% Cytofix (paraformaldehyde)/Cytoperm (BD Biosciences, San Jose, CA) diluted in PBS. Cells were rinsed in PBS and blocked with 0.1% Triton-X100 diluted in 10% FBS/PBS or blocked with Perm/Wash buffer (BD Biosciences) for 30 min at room temperature (RT) followed by a PBS rinse.

To verify neuronal phenotype, cells were incubated with primary antibodies specific for βIII-tubulin (TUJ1, Sigma Aldrich, St. Louis, MO) diluted in PBS for 1 hr at 37°C. The cells were then rinsed with PBS prior to incubating the cells for 1 hr at 37°C with goat anti-mouse secondary antibody at 1:2000 (Alexa Fluor 594, Invitrogen, Carlsbad, CA).

As a marker for chlamydial inclusions, cells were incubated for 1 hr at 37°C at 1:10 with a directly conjugated, FITC-anti-*Chlamydia *antibody which also contained Evans Blue for staining the cytoplasm (IMAGEN, Oxoid Ltd., Hampshire, UK) or 1:50 with FITC-anti-*Chlamydia *antibody (60C19, Fitzgerald, Concord, MA).

Colabeling for active caspase and chlamydial inclusions was accomplished as follows. Cells were incubated with a directly conjugated, FITC-anti-*Chlamydia *antibody (61C75, Fitzgerald) at 1:50 for 1 hr at 37°C, and incubated at 1:200 for 1 hr at 37°C with rabbit anti-cleaved caspase-3 (Cell Signaling Technology, Inc., Danvers, MA). A goat anti-rabbit secondary antibody at 1:2000 (Alexa Fluor 594, Invitrogen) was used to detect the caspase labeling. The slides were coverslipped using Prolong^® ^Gold anti-fade reagent with DAPI (Invitrogen).

For visualization of nuclear profiles in apoptosis analysis, Hoechst dye 33258 (Sigma-Aldrich, St. Louis, MO) was used at 1:1000.

Specificity of the secondary antibodies was confirmed by incubating with the FITC- or rhodamine-conjugated goat anti-mouse secondary without prior labeling by primary antibody.

### Annexin V-FITC Fluorescence

Infected and uninfected cells grown in chamber slides were rinsed in PBS and assayed for phosphatidylserine using the Annexin V-FITC Fluorescence Microscopy Kit (BD Biosciences, San Jose, CA) according to manufacturer's directions. Following annexin V labeling, the cells were fixed for 15 minutes in 1% Cytofix (BD Biosciences, San Jose, CA) diluted in 1× Annexin V Binding Buffer. The slides were coverslipped using Prolong^® ^Gold anti-fade reagent with DAPI (Invitrogen).

### Immunohistochemistry

AD and non AD control brain tissues (Late-onset AD and age-matched control brains obtained from Medical College of Pennsylvania/Hahnemann/Drexel University School of Medicine) were deparaffinized through xylenes and graded alcohols (100% - 70%) followed by a distilled water rinse. The slides were then washed with PBS, pH 7.4 and incubated in 3% hydrogen peroxide for 5 min followed by a PBS rinse. Samples were incubated in Citra antigen retrieval buffer (BioGenex, San Ramon, CA) in a microwave for 30 sec, followed by rinsing in PBS. Samples were blocked over 30 min in 2% fetal bovine serum (FBS) diluted in PBS followed by incubation for 1.5 hr at 37°C in an anti-chlamydial antibody at 1:200 (Biodesign Inc., Carmel, NY) diluted in PBS. Subsequently, samples were rinsed with PBS followed by incubation in a goat anti-rabbit antibody conjugated to alkaline phosphatase red at 1:400 for 1 hr at 37°C. Samples were washed with PBS. Samples were counterstained with Harris's Hematoxylin and then rinsed in ddH_2_O and then PBS for 5 min. Slides were dehydrated in graded alcohols (70–100%) and Xylenes and cover-slipped using Permount.

### Caspase Assay

Activity of Caspase-3/7 in uninfected and infected SK-N-MC cells was assayed with the Apo-ONE Homogeneous Caspase-3/7 Assay (Promega, Madison, WI) according to manufacturer's directions. 10^5 ^cells were incubated in growth media with either 1 μM staurosporine to induce apoptosis or with the DMSO vehicle for 4 hr at 37°C in 5% CO_2_. The cells were washed with PBS then brought up to 1 ml final volume in PBS. The vials were frozen at -20°C to ensure cell lysis. After the cells were thawed, 100 μl aliquots (1 × 10^4 ^cells) were pipetted into a 96-well plate with optical bottom (Nunc 165305) and the Apo-ONE reagent was added at 1:1 to each well; each condition was tested in triplicate by loading 3 wells with the same materials. The plate was gently mixed for 30 seconds then placed in the plate reader (Fluoroskan Ascent CF; Thermo Labsystems, Philadelphia, PA) for fluorescence measurement at excitation and emission wavelengths of 490 nm and 520 nm, respectively. Fluorescence readings were taken at 30 min intervals for 5 hours of reaction time, during which fluorescence increased monotonically from the first measurement to the last. Enzymatic activity for each well was analyzed as the maximal rate of substrate cleavage calculated from the assay's rate of fluorescence increase; the greatest slope occurred 2 to 4 hr after initiating the assay for all samples. To facilitate interpretation of the data, the activities were normalized to the change in activity induced by staurosporine in the uninfected cells. The experiment was performed on 3 cultures, yielding 3 plates for 24, 48 and 72 hour post-infection time points.

### Image Capture

Slides were viewed on a Nikon E800 epifluorescence microscope. Images were captured with a Spot RT camera (Diagnostic Instruments, Sterling Heights, MI) and analyzed using Image Pro Plus 4.5 software (Media Cybernetics, Bethesda, MD).

## Authors' contributions

All authors have read and approved the final manuscript.

DA: conceived of the study and participated in its design and coordination and in drafting the manuscript;

MR: tissue culture and immunocytochemistry;

DW: tissue culture, immunocytochemistry and enzyme assays;

MB: enzymatic and statistical analyses and participated in drafting the manuscript;

EA: tissue culture and immunocytochemistry;

CH: immunohistochemistry;

BB: participated in the studies design and coordination and in drafting the manuscript.
